# Feasibility of cortical bone trajectory screws for bridging fixation in revision surgery for lumbar adjacent segment degeneration

**DOI:** 10.1097/MD.0000000000026666

**Published:** 2021-07-16

**Authors:** Long Wang, Yong-Hui Zhao, Xing-Bo Cai, Jin-Long Liang, Hao-Tian Luo, Yu-Long Ma, Yong-Qing Xu, Sheng Lu

**Affiliations:** a Postgraduate College of Kunming Medical University, No. 1168 Chunrong Xi Road, Kunming, China; b Department of Orthopedics, 920th Hospital of Joint Logistics Support Force, 212 Daguan Road, Kunming, China; c Department of Orthopedics, The First People's Hospital of Yunnan Province, The Affiliated Hospital of Kunming University of Science and Technology, The Key Laboratory of Digital Orthopedics of Yunnan Province, No. 157 Jinbi Road, Kunming, China.

**Keywords:** adjacent segmental disease, cortical bone trajectory screw, lumbar vertebrae, pedicle screw, revision

## Abstract

**Background:**

To investigate the feasibility of using cortical bone trajectory (CBT) screws for bridging fixation in revision surgery for lumbar adjacent segment degeneration and to provide a reference for clinical practice.

**Methods:**

Computed tomography scans of the lumbar spines of 36 patients in our hospital were used. Sixteen males and 20 females with an average age of 65.5 ± 10.5 years (range: 46 to 83 years) were included. Three-dimensional reconstruction was performed using computer software. Screws with appropriate sizes were selected for the L1 to L5 vertebral segments, and traditional pedicle screws were placed using the standard method. After completing screw placement, simulated placement of CBT screws was performed separately. No overlap occurred between the two screws in the process of CBT screw placement, and the placement point and direction were adjusted until screw placement completion. After all screw placement simulations were complete, according to the contact area of the cortical bone of the screw trajectory and the screw puncture position and distance through the trajectory, the screw placement results were categorized as excellent, good, general, and failure. Excellent and good ratings were considered successful, while a general rating was regarded as acceptable. Then, the success rate and acceptable rate of each segment of the lumbar spine were calculated.

**Results:**

Three hundred and sixty screw placement simulations were performed in lumbar pedicles, and 72 CBT screws were implanted in each vertebral body of the lumbar spine. The success rates in the L1 to L5 segments were 73.6%, 80.6%, 83.3%, 88.9%, and 77.8%, respectively, and the acceptable rates were 91.7%, 97.2%, 97.2%, 100%, and 91.7%, respectively. The overall success rate and acceptable rate of CBT screw placement in the lumbar spine were 80.8% and 95.6%, respectively.

**Conclusion:**

CBT screws are feasible for bridging fixation in lumbar adjacent segment degeneration revision surgery, and the accuracy of screw placement in different lumbar vertebrae varies.

## 1 Introduction

In recent years, cortical bone trajectory (CBT) screws have constituted a new method of posterior lumbar internal fixation. This technique not only can be used in patients with osteoporosis but also has the advantages of minimal trauma, strong holding power, and few complications.^[1–6]^ CBT screw technology has gradually increased in clinical applications and has achieved satisfactory results in lumbar spondylolisthesis and degenerative diseases.^[7–9]^ CBT screws can be used in combination with traditional pedicle screws. Cortical bone channel screws are used for the upper vertebral body, pedicle screws are used for the lower vertebral body, and pedicle screws are used on one side, while cortical bone channel screws are used on the other side.^[10–12]^ In the literature, cortical bone channel screws and pedicle screws have been reported to be inserted simultaneously in the same pedicle to improve fixation strength.^[13]^ In addition, CBT screws can also be used for bridging and fixation in revision surgery for diseases of the lumbar vertebrae adjacent to the vertebral body. In this way, bridging and fixation can be completed without removing the original internal fixation device, which never simplifies the operative steps, minimizes trauma, or promotes patients’ recovery. However, few reports on this method are available, and the operation is difficult. What is the accuracy of CBT screw placement for bridging fixation? Do various cones differ? At present, related anatomical research is lacking. In this study, we use digital technology to study the feasibility of CBT screws in the repair and bridging fixation of lumbar vertebra-adjacent vertebral body disease and provide a reference for clinical application.

## 2 Materials and methods

This study was conducted in accordance with the Declaration of Helsinki and approved by the institutional review board of the 920 Hospital of the joint service support force of the Chinese people's liberation army. The need for informed consent was waived by the institutional review board.

### 2.1 General data

The computed tomography (CT) data of 36 inpatients from December 2015 to June 2018 were obtained, including 16 males and 20 females with an average age of 57.90 ± 17.40 years (range: 46–83 years). Inclusion criteria:

(1) patients aged greater than 18 years; and
(2) complete lumbar CT scan data.

Exclusion criteria:

(1) lumbar deformity or vertebral dysplasia;
(2) damaged vertebral structures due to fractures, infections, or tumors; and
(3) a history of lumbar surgery.

### 2.2 Study methods

**Figure 1:**
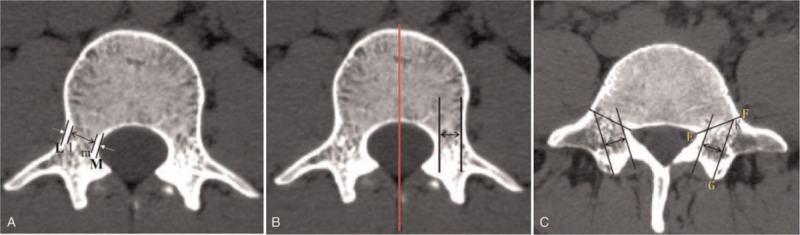
(A) A plain scan with the widest lateral wall of the pedicle is first selected, and then the narrowest width in the cross-section is selected. Two parallel lines are drawn along the pedicle axis (L and M) at lateral edge of the lateral wall and at the medial edge of the medial wall of the pedicle. The distance between lines of L and M is the PW. Two parallel lines are drawn along the pedicle axis (l and m) at the medial edge of the lateral wall and at lateral edge of medial wall of the pedicle. The distance between lines l and m is the SBW. (B) CBT width in L1-L4 pedicles. A plain scan with the widest lateral wall of the pedicle is selected. Straight lines are created parallel to the mid-line of the vertebral body at the medial edge of the lateral wall and the lateral edge of the medial wall of the pedicle to measure the distance between the two lines. (C) Schematic of the TRW of the L5 pedicle. A line is drawn at the narrowest width of this plane (line EF). This line intersects the medial edge of the lateral wall at point K. Then, the lateral ridge of the lamina is identified and marked as point G. A line parallel to the line GK is drawn at the lateral edge of the medial wall. Finally, the distance between two parallel lines is measured. SBW = pedicle spongy width, TRW = trajectory reference width.

The lumbar CT scan data from 36 patients were imported into Mimics 19.0 software for three-dimensional (3D) reconstruction in Digital Imaging and Communications in Medicine format. Since the height of the L1-L5 pedicles is greater than the width, the pedicle width is the main factor limiting the diameter of a screw. Therefore, we first measured the pedicle width (PW), pedicle spongy width, and pedicle trajectory reference width (TRW), as shown in Figure 1. Among these parameters, PW and pedicle spongy width are the references for selecting the traditional pedicle screw diameter, TRW is the main reference for selecting the CBT screw diameter, and the CBT screw diameter should be ≤ TRW. Combined with the pedicle parameters and considering the placement of both CBT screws and traditional pedicle (TP) screws in the same pedicle, we selected screws with appropriate diameters based on the relevant parameters measured (Table 1).

**Table 1:** Related parameters and diameters of the screws in simulated placements.

Lumbar	Related parameters of pedicle trajectory (mm)			Screw diameter (mm)	
segment	TRW	PW	TRW	TP	CBT
L1	4.6 ± 1.1	7.7 ± 1.3	4.1 ± 1.1	5.5	4.0
L2	5.8 ± 1.2	8.7 ± 1.5	4.7 ± 0.9	6.0	4.0
L3	7.2 ± 1.1	10.1 ± 1.2	5.8 ± 0.9	6.5	4.5
L4	9.2 ± 1.4	12.4 ± 1.5	7.1 ± 1.2	6.5	5.0
L5	15.9 ± 1.7	6.6 ± 1.0	6.6 ± 1.0	6.5	5.0

CBT = Cortical bone trajectory, PW = pedicle width, TP = traditional pedicle, TRW = trajectory reference width.

**Figure 2:**
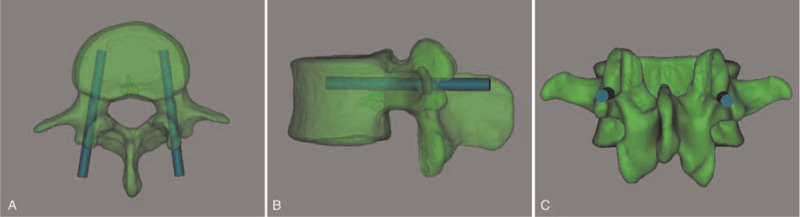
The criteria of the TP screw placement. (A) The screw entry point is located at the vertex point of the “λ”-shaped ridge. (B) The screw trajectory is close to the upper edge of the pedicle and parallel to the endplate of the vertebral body. (C) The screw is placed along the pedicle axis in the cross section, and the depth to anterior wall of the vertebral body. TP = traditional pedicle.

**Figure 3:**
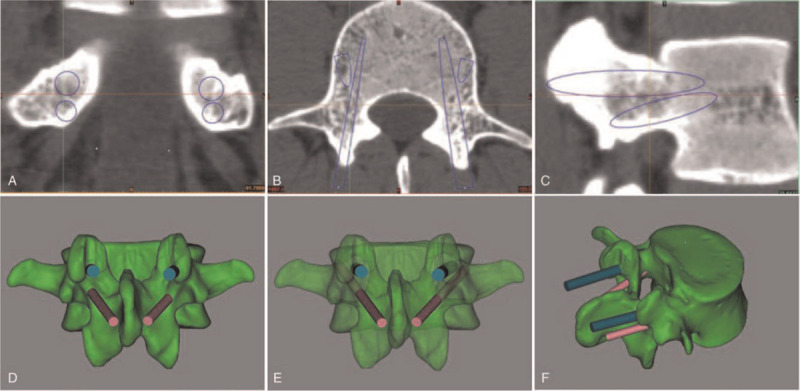
Placement of CBT screws with constant pedicle screw parameters: the location of the screw locus (A-C) in the coronal position, cross section and sagittal plane, and the location of screw placement point (D) of the two screws. Through transparence of the vertebral body, it can be judged that the two screws are not coincident (E). CBT screws pierce the medial wall of the vertebral arch root (F).

**Figure 4:**
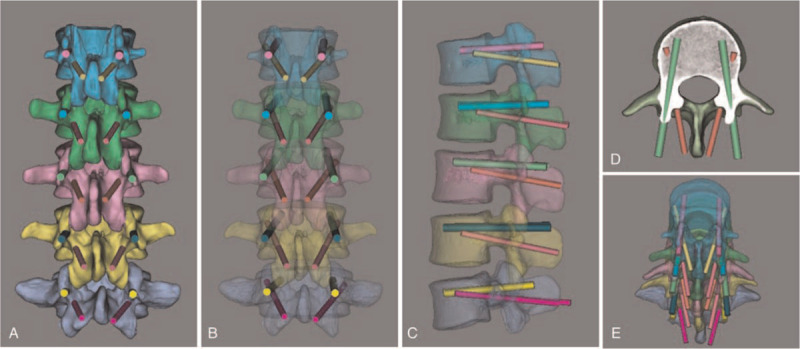
Schematic of the CBT screw placement. The TP screw is placed at the vertex of the “λ”-shaped ridge. The CBT screw placement site is located at the farther medial-inferior site (A). The anteroposterior and lateral view of the vertebral body (transparent mode) shows the positional relationship of the 2 screws (B/C). The axial view shows that the CBT screw is located under the pedicle screw, and the tip of the CBT screw reaches the sidewall of the vertebral body (D). The top view shows the positional relationship of the 2 screws (E).

First, TP screws were positioned in the L1 to L5 pedicles (Fig. 2). The criteria for screw placement are described below. (1) The vertex of the “λ”-shaped ridge was chosen as the entry point for screw placement. (2) Regarding the direction of screw placement, the screw trajectory in the sagittal plane was close to the upper edge of the pedicle and parallel to the endplate of the vertebral body. The screw was placed along the pedicle axis in the cross section, and the depth to the anterior wall of the vertebral body. (3) The diameter of the screw was 5.0 mm in L1, 6.0 mm in L2, 6.5 mm in L3, 6.5 mm in L4, and 6.5 mm in L5. After TP screw placement, the TP pedicle screw parameters were unchanged, and CBT screw placement was simulated. The diameters of the CBT screws were 4.0 mm in L1, 4.0 mm in L2, 4.5 mm in L3, 5.0 mm in L4, and 5.0 mm in L5. According to the characteristics of the CBT screw and the position of the pedicle screw, the direction of CBT screw placement was adjusted in the cross section, coronal plane, and sagittal plane by using Mimics 19.0 software to avoid overlap between the two screws (Fig. 3), and all CBT screws were successively inserted according to the requirements (Fig. 4). In the process of screw placement, the position of the puncture cortex, the distance of screw penetration, and the contact area of the screw track were recorded for grading and evaluation. Grading standard: Grade I: CBT screws are completely wrapped by cortical bone, and the screw track passes through at least three cortical areas (the dorsal cortex, medial wall of the pedicle, lateral wall of the pedicle, and lateral wall of the vertebral body); grade II: CBT screws pass through < 2 mm of the cortex, and the screw track passes through at least three cortical areas; grade III: screws pass through ≥ 2 mm and < 4 mm of the cortex, and the screw track passes through at least three cortical areas; grade IV: the screw passes through ≥ 4 mm of the cortex, or the screw track passes through two or fewer layers of cortex. Grade I is excellent; grade II is good for penetrating the outer wall of the pedicle or the outer wall of the cone and is acceptable for penetrating the inner wall or the lower wall of the pedicle; grade III is acceptable for penetrating the outer wall of the pedicle or the outer wall of the cone but is unacceptable for penetrating the inner wall or the lower wall of the pedicle; and grade IV corresponds to failed screw placement. Finally, the success rate ((excellent + good) / total number of screws), acceptable rate ((excellent + good + general) / total number of screws), and failure rate (failure / total number of screws or 1 - acceptability) were calculated to evaluate the accuracy of screw placement.

### 2.3 Statistical analysis

SPSS 22 statistical software (SPSS) was used for statistical analysis. The Kolmogorov-Smirnov test was used to assess whether the quantitative data conformed to a normal distribution. Quantitative data with a normal distribution are presented as $\bar{x}\;\;\pm \;\text{s}$ and were compared using an independent sample *T* test, including the parameters of the left and right pedicles. *P* < .05 was considered statistically significant.

## 3 Results

In this study, 360 screw placement simulations were performed in lumbar pedicles, and 72 CBT screws were implanted in each vertebral body of the lumbar spine. L1 pedicle screw results: 33 screws were excellent, 20 were good, 12 were general, and two failed; the success rate of screw placement was 80.6%, the acceptable rate was 97.2%, and the failure rate was 2.8%. L2 pedicle screw results: 44 screws were excellent, 14 were good, 12 were general, and two failed; the success rate of screw placement was 80.6%, the acceptable rate was 97.2%, and the failure rate was 2.8%. L3 pedicle screw results: 44 screws were excellent, 16 were good, 10 were general, and two failed; the success rate was 83.3%, the acceptable rate was 97.2%, and the failure rate was 2.8%. L4 pedicle screw results: 50 screws were excellent, 14 were good, eight were acceptable, and 0 failed; the success rate of screw placement was 88.9%, and the acceptable rate was 100%. L5 pedicle screw results: 38 screws were excellent, 18 were good, 10 were general, and six failed; the success rate was 77.8%, the acceptable rate was 91.7%, and the failure rate was 8.3%. The success rate of lumbar spine screw placement was 80.8%, the acceptable rate was 95.6%, and the failure rate was 4.4% (Table 2).

**Table 2:** CBT screw placement results.

Lumbar	Screw grade					Screw setting results (%)		
vertebra	Excellent	Good	General	Fail	Sample	Success	Acceptable	Failure
L1	33	20	13	6	72	73.60%	91.70%	8.30%
L2	44	14	12	2	72	80.60%	97.20%	2.80%
L3	44	16	10	2	72	83.30%	97.20%	2.80%
L4	50	14	8	0	72	88.90%	100%	0.00%
L5	38	18	10	6	72	77.80%	91.70%	8.30%
Total	209	82	53	16	360	80.80%	95.60%	4.40%

## 4 Discussion

With the aging of the world population and the development of the pedicle screw technique, the number of patients undergoing lumbar fusion with internal fixation has increased rapidly each year. Between 1994 and 2006, the National Survey of Ambulatory Surgery revealed 5.4- and 2.6-fold increases in the number of surgeries for patients with intervertebral disc disease and spinal stenosis, respectively, and the number of patients requiring intervertebral fusion surgery increased by 340%.^[14]^ Deyo ^[15]^ analyzed the National Health Data from the online Health Care Utilization Project and found that the number of lumbar fusion surgeries conducted between 1996 and 2011 increased by 600%. However, spinal fusion changes the biomechanical environment of the spine and stresses the intervertebral disc and facet joints of adjacent segments, thereby accelerating degeneration of adjacent segments and resulting in secondary lumbar disc herniation, lumbar spinal stenosis, and degenerative lumbar spondylolisthesis. This phenomenon is called lumbar adjacent segment degeneration (LASD), and patients with severe symptoms must be treated with revision surgery.^[7,16,17]^ Different studies have reported that the incidence of LASD varies, and the incidence of LASD varies between studies. According to imaging evidence, the prevalence of LASD exceeds 40%, and the rate of surgical intervention ranges from 5.2% to 18.5%.^[7,8]^ LASD repair often requires removal of the original internal fixation device, resulting in a prolonged operative time, intraoperative blood loss, many postoperative complications, and increased difficulty and risks of surgery and thus leading to substantial challenges for many surgeons.^[18,19]^

The advent of the CBT screw provides a new option for fixation in LASD repair. CBT screws can be used for bridge fixation of adjacent vertebrae to achieve revision fixation, which reduces the technical difficulty and risk of traditional revision surgery. In 2014, Rodriguez et al^[20]^ used an O-arm navigation system to complete LASD repair in five patients by placing CBT screws in the pedicle of the fused upper vertebra without removing the original internal fixation device. During the follow-up of 10 to 15 months, no surgical complications occurred, symptoms were significantly improved, and interbody fusion was achieved. Wang et al^[21]^ also reported satisfactory results for 12 patients with lumbar spondylitis who underwent revision surgery with CBT screws without removing the internal fixation device. Chen et al^[22]^ performed revision surgery on six patients with LASD using a C-arm machine alone, and satisfactory results were also achieved. Based on these studies, CBT screws can be used for revision surgery in patients with LASD; however, related reports are rare in the literature. Due to differences in the pedicle anatomy of different segments of the lumbar spine, the same lumbar segment displays some differences between individuals. Can CBT screws be used for revision bridge fixation in patients with LASD? What is the accuracy rate of lumbar revision surgery? The use of CBT screws in bridge fixation to complete revision surgery has been challenging in an increasing number of patients with LASD.

Mullin et al^[9]^ selected lumbar CT scan data from 47 patients and divided them into two groups according to their history of lumbar surgery. In one group, CBT screws were placed based on the locations of the original pedicle screws. In the other group, two types of screws were placed concurrently, and the authors reported the feasibility of inserting two screws at the same time. However, the results obtained from the study have very limited value because of the nonuniform distribution of the surgically fixated segments in the group with a surgical history, namely, only three patients underwent original fixation in L1. In the present study, we used digital technology to simulate CBT screw placement based on TP screws and verified the feasibility of CBT screw placement. Using this approach, we successfully solved the problem of the “nonuniform distribution of surgically fixated segments” in the study by Jeffrey et al.

In the present study, lumbar CT scan data from 36 patients were used to simulate screw placement after 3D reconstruction. According to the parameters of the pedicle, the diameter of the selected CBT screw was 4.0 to 5.0 mm, and the diameter of the pedicle screw was 5.5 to 6.5 mm. These screw sizes were not the same as those selected in previous studies. Ueno et al^[13]^ used CBT screws with a diameter of 4.5 mm (L1-L5: 4.5 mm) and TP screws with a diameter of 5.5 to 6.5 mm (L1-L2: 5.5 mm and L3-L5: 6.5 mm). Rodriguez et al^[20]^ selected CBT screws with a diameter of 5.5 mm and TP screws with a diameter of 7.0 to 7.5 mm (L1, L2, and L4: 7.0 mm; L3 and L5: 7.5 mm) for revision surgery. Different screw sizes might have different effects on the success rate and biomechanical strength. Screws that are too large might also increase the risk of fracture of the pedicle isthmus. Thus, further studies are needed. Based on conventional criteria, we simulated TP screw placement and then CBT screw placement. This study has good repeatability and had a sufficient sample size. At the same time, the screw trajectory, the cortical bone puncture, and the distance to the screw's exit point can be observed intuitively, and the result of screw placement is simple to judge. When evaluating screw placement, we classified screw placement according to the contact area of the CBT screw locus and the distance between the screw and the cortex. Then, the results of screw placement were classified as excellent, good, general, and failure and combined with the location of the puncture path (different surgical risks for a different area). Excellent and good ratings were considered successful, and a general rating was regarded as acceptable, and then the success rate and acceptable rate of each segment of the lumbar spine were calculated. The evaluation method fully considers the characteristics of the CBT screw track and the safety of screw placement such that the evaluation of screw placement results is more reasonable and scientific. The results showed that the success rate of lumbar spine screw placement was 80.8% (73.6% ∼ 88.9%), and the acceptable rate was 95.6% (91.7% ∼ 100%). Some differences in the success rate and acceptable rate of screw placement were found in different segments of the lumbar spine. The accuracy rate of screw placement from high to low was L4 > L3 > L2 > L5 > L1, which is related to the screw size and anatomical structure of the pedicle.

This design has some shortcomings because pedicle screws are placed in accordance with conventional standards, but in clinical practice, pedicle screws can be located in the upper, middle, or lower walls of the pedicle in the sagittal position and in the outer, middle, or inner walls of the pedicle in the coronal position. In the latter study, we classified pedicle screws according to the different positions of the pedicle screw in the pedicle and further improved the accuracy of screw placement. Because the operative technique, pedicle screw position, and individual differences in patients affect the results of screw placement, some differences in screw placement simulations in the software will occur. In addition, we explored only the feasibility of using CBT screws to bridge and fix vertebral bodies with upper and lower fixation with respect to posterior fixation for LASD repair. In addition to posterior fixation, the curative effect of LASD revision is also related to interbody fusion and nerve root decompression. A computer navigation system can be used to facilitate screw placement in clinical practice. Based on the CBT screw channel, we can also design a guide plate to assist with screw placement. When conditions are not sufficient, we can reconstruct 3D images according to patients’ initial CT data, simulate screw placement according to the position and size of the fixed screws, and then simulate CBT screw placement based on the pedicle screws again to provide a reference for the operation.

## 5 Conclusions

In this study, CBT screws were feasible for bridging fixation in LASD revision surgery, and the accuracy of screw placement accuracy in different lumbar vertebrae varied. However, the feasibility of using different pedicle screw positions requires further exploration. In addition, further experimental research and clinical application verification are needed.

## Author contributions

**Conceptualization:** Long wang, Yonghui Zhao, Sheng Lu.

**Data curation:** Long wang, Yonghui Zhao, Xingbo Cai.

**Formal analysis:** Yonghui Zhao, Xingbo Cai, Jinlong Liang.

**Funding acquisition:** Yongqing Xu, Sheng Lu.

**Investigation:** Yonghui Zhao, Xingbo Cai, Yulong Ma.

**Methodology:** Long wang, Yonghui Zhao, Haotian Luo, Sheng Lu.

**Writing – original draft:** Yonghui Zhao.

**Writing – review & editing:** Long wang, Sheng Lu.

## References

[1] Santoni BG Hynes RA Mcgilvray KC. Cortical bone trajectory for lumbar pedicle screws. Spine J 2009;9:366–73. 18790684 10.1016/j.spinee.2008.07.008

[2] Matsukawa K Yato Y Imabayashi HA Hosogane N Asazuma T Nemoto K. Biomechanical evaluation of the fixation strength of lumbar pedicle screws using cortical bone trajectory: a finite element study. J Neurosurg Spine 2015;23:471–8. 26161515 10.3171/2015.1.SPINE141103

[3] Kasukawa Y Miyakoshi N Hongo M Ishikawa Y Kudo D Shimada Y. Short-term results of transforaminal lumbar interbody fusion using pedicle screw with cortical bone trajectory compared with conventional trajectory. Asian Spine J 2015;9:440–8. 26097661 10.4184/asj.2015.9.3.440 PMC4472594

[4] Marengo N Berjano P Cofano F. Cortical bone trajectory screws for circumferential arthrodesis in lumbar degenerative spine: clinical and radiological outcomes of 101 cases [J]. Eur Spine J 2018;27: Suppl 2: 01–9. 10.1007/s00586-018-5599-8 29663147

[5] Matsukawa K Yato Y Nemoto O Imabayashi H Asazuma T Nemoto K. Morphometric measurement of cortical bone trajectory for lumbar pedicle screw insertion using computed tomography. J Spinal Disord Tech 2013;26:E248–53. 23429319 10.1097/BSD.0b013e318288ac39

[6] Gautschi OP Garbossa D Tessitore E. Maximal access surgery for posterior lumbar inter body fusion (PLIF) with divergent, cortical bone trajectory (CBT) pedicle-screws: a good option for minimize spine access and maximize the field for nerve decompression. J Neurosurg Sci 2017;61:335–41. 26082380 10.23736/S0390-5616.16.03230-6

[7] Park P Garton HJ Gala VC Hoff JT McGillicuddy JE. Adjacent segment disease after lumbar or lumbosacral fusion: review of the literature. Spine 2004;29:1938–44. 15534420 10.1097/01.brs.0000137069.88904.03

[8] Chen G Bridwell KH Lenke LG. Adjacent segment disease following lumbar/thoracolumbar fusion with pedicle screw instrumentation: a minimum 5-year follow-up. Spine 2007;32:2253–7. 17873819 10.1097/BRS.0b013e31814b2d8e

[9] Mullin JP Perlmutter B Schmidt E Benzel E Steinmetz MP. Radiographic feasibility study of cortical bone trajectory and traditional pedicle screw dual trajectories. J Neurosurg Spine 2016;25:727–32. 27391396 10.3171/2016.4.SPINE151483

[10] Matsukawa K Yato Y Kato T Imabayashi H Asazuma T Nemoto K. Cortical bone trajectory for lumbosacral fixation: penetrating S-1 endplate screw technique. J Neurosurg Spine 2014;21:203–9. 24766288 10.3171/2014.3.SPINE13665

[11] Tortolani PJ Stroh DA. Cortical bone trajectory technique for posterior spinal instrumentation[J]. J Am Acad Orthop Surg 2016;24:755–61. 27755261 10.5435/JAAOS-D-15-00597

[12] Takenaka S Mukai Y Tateishi K Hosono N Fuji T Kaito T. Clinical outcomes after posterior lumbar interbody fusion. Clin Spine Surg 2017;30:E1411–8. 28266955 10.1097/BSD.0000000000000514

[13] Ueno M Imura T Inoue G Takaso M. Posterior corrective fusion using a double-trajectory technique (cortical bone trajectory combined with traditional trajectory) for degenerative lumbar scoliosis with osteoporosis[J]. J Neurosurg Spine 2013;19:600–7. 24010899 10.3171/2013.7.SPINE13191

[14] Best MJ Buller LT Eismont FJ. National trends in ambulatory surgery for intervertebral disc disorders and spinal stenosis: a 12-year analysis of the national surveys of ambulatory surgery. Spine 2015;40:10.1097/BRS.0000000000001109 26267820

[15] Deyo RA. Fusion surgery for lumbar degenerative disc disease: still more questions than answers. Spine J 2015;15:272–4. 25598279 10.1016/j.spinee.2014.11.004

[16] Madan SS Boeree NR. Comparison of instrumented anterior interbody fusion with instrumented circumferential lumbar fusion. Eur Spine J 2003;12:567–75. 14673717 10.1007/s00586-002-0516-5 PMC3467994

[17] Yu SW Yen CY Wu CH Kao FC Kao YH Tu YK. Radiographic and clinical results of posterior dynamic stabilization for the treatment of multisegment degenerative disc disease with a minimum follow-up of 3 years. Arch Orthop Trauma Surg 2012;132:583–9. 22262469 10.1007/s00402-012-1460-4

[18] Owens RK Djurasovic M Onyekwelu I Bratcher KR McGraw KE Carreon LY. Outcomes and revision rates in normal, overweight, and obese patients 5 years after lumbar fusion. Spine J 2016;16:1178–83. 27293121 10.1016/j.spinee.2016.06.005

[19] Ahn J Tabaraee E Bohl DD Aboushaala K Singh K. Primary versus revision single-level minimally invasive lumbar discectomy: analysis of clinical outcomes and narcotic utilization. Spine 2015;40:1025–30. 10.1097/BRS.0000000000000976 25955188

[20] Rodriguez A Neal MT Liu A. Novel placement of cortical bone trajectory screws in previously instrumented pedicles for adjacent-segment lumbar disease using CT image-guided navigation[J]. Neurosurg Focus 2014;36:E9. 10.3171/2014.1.FOCUS13521 24580010

[21] Wang YY Zhang F Fan SW Pulido-Rivas P Sola RG. Application of cortical bone trajectory screw in the revision of lumbar disease[J]. Chin J Orthop 2017;37:1143–9.

[22] Chen CH Huang HM Chen DC Wu CY Lee HC Cho DY. Cortical bone trajectory screws fixation in lumbar adjacent segment disease: A technique note with case series. J Clin Neurosci 2017;48:224–8. 29208475 10.1016/j.jocn.2017.11.008

